# Incorporating Scoring Risk Models for Care Planning of the Elderly with Chronic Kidney Disease

**DOI:** 10.1155/2017/8067094

**Published:** 2017-11-28

**Authors:** Josefina Santos, Isabel Fonseca

**Affiliations:** ^1^Nephrology Department, Hospital de Santo António, Centro Hospitalar do Porto (CHP), Porto, Portugal; ^2^Unit for Multidisciplinary Research in Biomedicine (UMIB), Abel Salazar Biomedical Sciences Institute (ICBAS), University of Porto, Porto, Portugal; ^3^EPIUnit, Institute of Public Health, Universidade do Porto (ISPUP), Rua das Taipas 135, 4050-600 Porto, Portugal

## Abstract

Mortality in chronic kidney disease remains high, particularly among the elderly, who represent the most rapidly growing segment of the end-stage renal disease population in wealthier countries. The management of older adults with chronic kidney disease has become a clinical challenge, and care for those patients expected to progress to end-stage renal disease should focus on evaluating the overall benefit of offering renal replacement therapy to them. Predictive mortality models may help to inform shared decision-making in the trajectory of the elderly with chronic kidney disease. This review discusses current literature on the available predictive models for predicting survival in elderly chronic kidney disease patients and reflects the author's own interpretation and experience.

## 1. Introduction 

The prevalence of chronic kidney disease (CKD) is higher in older people. Patients over 65 years of age represent the most rapidly growing segment of the end-stage renal disease (ESRD) population in wealthier countries [1, 2], as well as showing a high prevalence of earlier stages of CKD, with relative prevalence equally striking for populations in the USA, Canada, and Europe [1, 3–5].

The management of older adults with CKD has become a clinical challenge, and care for those patients expected to progress to ESRD should focus on evaluating the overall benefit of offering renal replacement therapy (RRT) to them. Although survival may have improved over time for older patients initiating dialysis [6], this survival benefit in the elderly is lower as compared to their younger counterparts [7]. Comparing dialysis versus conservative management, dialysis may be associated with a very small survival benefit [8] and associated with a concomitant overall decline in functional and cognitive status [9] and more hospitalizations [10].

In elderly patients with significant comorbidities, conservative management may therefore be a better therapeutic option, as dialysis is unlikely to prolong or improve quality of life [11].

Beyond the usual clinical criteria, the RRT decisions in elderly patients must incorporate assessment of physical and cognition function and other components of geriatric syndrome.

Recently there is a growing interest in developing predictive mortality models to improve patient outcomes through individualized risk prediction. These predictive models may help the nephrologist in their discussions with patients and their families about suitability or otherwise of initiating dialysis.

This review discusses current literature on the available predictive models for predicting survival in elderly CKD patients and reflects the author's own interpretation and experience.

## 2. Risk Prediction Models

### 2.1. Definition, Performance, and Validation

Risk prediction models are based on equations designed on the basis of prognostic factors and clinical outcomes, available at the time the prediction is made, and collected in specific and representative cohorts of individuals followed up for a given period of time [12, 13]. Each prognostic factor or variable is awarded a weight (coefficient) and combined in a mathematical formula, the so-called risk equation, to predict an outcome of interest [12, 13].

The performance of a risk prediction model is commonly assessed by testing its calibration and discrimination. Calibration describes the agreement of observed and predicted event rates [14], and discrimination expresses the ability of prediction model to distinguish individuals who will develop the outcome of interest from those who will not [15].

The most used measure of discrimination is the C-statistic, a measure of concordance between model-based risk estimates and observed events [16–18]. The C-statistic ranges from 0.5 (random concordance) to a theoretical maximum of 1 (perfect concordance), but it has several limitations [18].

First, as a single number, it summarizes the discrimination of a model but does not communicate all the information and lacks direct clinical application. The C-statistic does not effectively balance misclassification errors, and a weighted sum of sensitivity and specificity have more clinical relevance (predicting an individual who ultimately experiences an event to be at low risk; predicting an individual who does not experience an event to be at high risk), according to the principles of decision analysis [19]. Secondly, the value of the C-statistic depends not only on the model being assessed, but also on the distribution of risk factors in the sample to which it is applied.

Calibration, the agreement of observed and predicted outcomes, is most appropriately assessed using a calibration plot [16], which assesses how accurately the model's predictions match overall observed event rates. Unfortunately, in the majority of the studies calibration measures are often omitted [20, 21].

Another important question for physicians to consider is whether the score accurately predicts outcomes in people like their patients. A simple internal validation, that is, a computing performance measures in the same cohort that has been used to develop the model, usually leads to overoptimistic estimates of the performance of a prediction model [13].

Thus, the use of methods such as cross-validation has been proposed for assessing internal validity. With this method, the original cohort is split into a development and a validation sample, to develop the score in one group and test in the other [13, 22].

Another method that can be used if the number of individuals in the cohort is relatively low or to avoid false results caused by one particular random split is the bootstrapping based on many repeated splits of the data [13, 16, 22].

Even with a good performance measures achieved in the same cohort as the one that was used to develop the model, before adopting a risk score into practice, clinicians need to decide whether the score accurately predicts outcomes in people like their patients. Therefore, ideally, the model needs to be tested in a group of people that was not used to develop the model; it needs to be externally validated [23]; that is, the performance of the prediction model is tested in patients with the same disease but belonging to a different source population.

### 2.2. Clinical Usefulness

Clinical usefulness may be evaluated by utility and usability. The utility reflects the extent to which the risk score actually affects clinical decisions [24]. The usability reflects the availability of a clinical decision aid, such as a nomogram or online calculator, which would allow risk prediction at the bedside [21]. For a risk model to be useful in practice, it needs to include variables that are well defined, measurable, and readily available. Finally, information on outcomes based on these models must be transferred in a way that is understandable for all involved in shared decision-making process.

## 3. Risk Prediction Scoring Models for Elderly CKD Patients

### 3.1. Mortality Risk Prediction Models in CKD

In 2013, Tangri et al. [21] conducted a systematic review to identify prediction models for kidney failure, cardiovascular events, and all-cause mortality in CKD patients. They found five studies (6 models) [25–29] that examined either all-cause mortality or the composite outcome of kidney failure or death. More recently, Stryckers et al. [30], in an attempt to construct an algorithm that helps in planning the care of elderly people with advanced CKD, identified 4 risk prediction models that target elderly people with CKD 3–5 [28, 31, 32] and 12 models developed in elderly with ESRD [33–38].

Looking to the risk models that specifically included elderly people with CKD, the study of Johnson et al. [28] used the same variables (age, sex, eGFR, diabetes, hypertension, and anemia) for both outcomes (RRT or death). They found that although the same six variables predicted mortality (C-statistic 0.70) and its composite end of RRT and death (C-statistic 0.71), the overall prediction was markedly less effective than for RRT (C-statistic 0.91). They also found an inverse association between age and hypertension for death and a direct association for kidney failure. They concluded that predicting RRT requires a separate risk score, because predicting the composite endpoint would favor characteristics that predict mortality, since mortality is much more common than RRT in elderly patients. These models [28] were not validated externally, and calibration measures were not reported.

More recently, Bansal et al. [31] developed a prediction equation for 5-year risk of mortality for older people with CKD stages 3–5 not treated with dialysis. The equation included nine readily available clinical variables (age, sex, race, eGFR, urine albumin-to-creatinine ratio, smoking, diabetes mellitus, and history of heart failure and stroke), and it was externally validated in a large cohort of elderly CKD patients. This model has an acceptable calibration and discrimination in both the development (C-statistic = 0.72; 95% confidence interval, 0.68 to 0.74) and validation cohort (C-statistic = 0.69; 95% confidence interval, 0.64 to 0.74). However, one of the limitations pointed is that the validation cohort did not fits the frailty phenotype associated with CKD [39], because the authors enrolled well-functioning men and women, and it has been well established that frailty is an additional risk factor for mortality in CKD patients [31].

In another model, Weiss et al. [32] developed a risk prediction model in a retrospective cohort of patients aged 65 to 79 and 80 and older with moderate-to-severe CKD (eGFR, <30 mL/min per 1.73 m^2^) to predict mortality at 6 months and at 2 years. The model included sixteen comorbidities and measures of health and functional status. Although the C-statistics for each model for both periods (6 months and at 2 years) indicated a moderate discrimination (0.68–0.69), once more, this score risk was not externally validated. In addition, the presence of comorbidities was determined within administrative databases, and in retrospective data, which can considerably reduce the predictive performance of the model.

However, one of the strengths of Weiss et al. score [32] was the incorporation of nondisease specific measures including markers of healthcare use (e.g., hospitalizations) and functional status (e.g., falls, dementia), contrary to the other available risk prediction models for mortality in adults with CKD that mainly focus on traditional risk factors.

### 3.2. Mortality Risk Prediction Models in ESRD

In 2009, Couchoud et al. [34], using just clinical features, based on the REIN (French Renal Epidemiology and Information Network) cohort data, predicted 6-month mortality in elderly (aged 75 and older) with ESRD patients after initiating dialysis.

Nine risk factors were selected (demographic and baseline clinical variables), and the score showed good calibration, as reflected by the concordance between observed and expected mortality rates in the validation sample, but with only a moderate discrimination (mean C-statistic 0.70). The authors pointed out some limitations, namely, a selection bias due to the imputed missing data, and the fact that no information was available about ESRD patients who were not referred to nephrologists or did not receive dialysis. Therefore, this score cannot be generalizable to the entire population of elderly ESRD patients, particularly to the patients with high comorbidities and poor conditions, in which this score cannot replace the clinical judgment. This score can be used to facilitate discussion with patients and their families, but not to withhold dialysis [34].

The Couchoud et al. model [34] was further externally validated in an US population [33], although investigators modified the score and they concluded that indices performed poorly with respect to prediction of 6-month mortality in in older patients with ESRD commencing dialysis.

Since mortality may be high in the first few months after initiating dialysis, in 2015, in an attempt to improve their previous prognostic score [34], using the REIN registry, Couchoud et al. [35] chose to focus on very early mortality during the first 3 months of dialysis, in patients aged 75 years and older. They founded that male gender, age over 85 years, congestive heart failure, severe peripheral vascular disease, dysrhythmia, severe behavioral disorders, active malignancy, serum albumin, and impaired mobility were independently associated with 3-month mortality.

Despite a good calibration and discrimination, this model [35] had some limitations. First, this score was built within administrative databases. Second and more important, it was derived from patient population who have initiated RRT and do not include those who refuse, are not selected for, or do not survive to dialysis initiation.

Also, focused in early mortality after dialysis initiation (3 months), Thamer et al. [37], using the US Renal Data System (patients aged ≥ 67 years), validated a score and proposed a simple risk assessment questionnaire, based on ready available information (age, low albumin, assistance with daily living, nursing home residence, cancer, heart failure, and hospitalization).

This model [37] was not externally validated and only used data from administrative databases, with no inclusion of more detailed clinical and psychosocial data, which is much important in elderly ESRD patients. Moreover, this model excluded patients who did not choose dialysis and only included patients with a 2-year previous follow-up.

Floege et al. [40] have published another risk prediction model developed in European hemodialysis cohort with a mean age of 64 years old, using objective measurements only (i.e., no surprise question or dementia). This model was then validated in an external cohort of the Dialysis Outcomes and Practices Patterns Study (DOPPS) and exhibited a moderate discrimination (C-statistic of 0.68 to 0.79).

Nevertheless, the Floege et al. score [40] has not been developed nor validated in a cohort of elderly dialysis patients. In addition, because the development cohort includes only patients who survived the first 3 months, whereas the validation cohort of DOPPS includes mainly prevalent patients, it is still not a perfect risk predictor for frail elderly, in which the risk of short-term mortality is what needs to be predicted.

Considering the impact of comorbidity for predicting survival in elderly dialysis patients, Liu et al. [36] modified the original Charlson Comorbidity Index (CCI) [41] and developed a new comorbidity index (nCI) for mortality analyses for dialysis patients using administrative data, based on the comorbid conditions used by the United States Renal Data System (USRDS). The index was developed using the 2000 US incident dialysis population and validated using the 1999 and 2001 US incident dialysis populations and the 2000 US prevalent dialysis population. Interestingly, the Liu et al. comorbidity index [36] includes 11 comorbid conditions (atherosclerotic heart disease, congestive heart failure, cerebrovascular accident/transient ischemic attack, peripheral vascular disease, dysrhythmia, other cardiac diseases, chronic obstructive pulmonary disease, gastrointestinal bleeding, liver disease, cancer, and diabetes), but not the age factor, one of the components of the original CCI. The authors [36] showed that nCI performance was almost identical to the individual comorbid conditions regarding model fit, predictive ability, and effect on inference, and it its results showed that nCI is a better predictor than the CCI [41]. Actually, age and comorbidities should both be integrated in a risk prediction tool as major drivers for mortality.

Cohen et al. [42] developed a prognostic model to determine the risk of death in dialysis patients by combining selected variables from the modified CCI (age, presence of dementia, and peripheral vascular disease) and serum albumin with the nephrologist's answer to the Surprise Question (“Would I be surprised if this patient died within the next 6 months?”). This simple bedside tool for predicting 6-month mortality had superior prognostic value than either tool independently. Although this prognostic model has not been developed or validated in elderly dialysis patient, it has the advantage of being available as online calculator (“Surprise Question Predictor”at http://nephron.com).

However, we can argue that this model [42] has the limitations of being based on subjective parameters (i.e., dementia), difficult to define in a dialysis patients. In addition, the Surprise Question is highly subjective and variable based on nephrologist training and knowledge of patient.

Recently, Wick et al. [43] developed a risk score (Alberta score) that potentially could be used to estimate mortality risk during the next 6 months for older patient initiating dialysis.

They identify several independent predictors of mortality, which include age of 80 years or older, early dialysis therapy, atrial fibrillation, congestive cardiac failure, lymphoma, metastatic cancer, and hospitalization in the prior 6 months.

They used a large population-based data source (renal registry data from Alberta, Canada) consisting of incident hemodialysis and peritoneal dialysis patients in outpatient settings, which should minimize selection bias.

The incorporation of variables like lymphoma and metastatic malignancy as mortality predictors; it maybe will add clinical utility in contexts in which these conditions appear with reasonable frequency. Moreover, hospitalizations in the 6 months prior to dialysis initiation, like in Thamer score [37], were also a mortality predictor and probably related to comorbidity and disease severity.

Although the Alberta score [43] seems to be a rigorously and useful derived model, it needs to be replicated in an independent population.

Finally, the Study of Heart and Renal Protection (SHARP) CKD-CVD model [44] was developed using data from 9270 patients with moderate-to-severe CKD (including CKD 3B, 4, 5, dialysis, and kidney transplant patients) in the Study of Heart and Renal Protection (SHARP) [45], followed for an average of 5 years. This model projects lifetime cardiovascular event risks, kidney disease progression, and (quality-of-life adjusted) survival. Higher age, previous cardiovascular events, and advanced CKD were the main contributors to increased individual disease risks. The model [44] performs well in categories of patients by CKD stage in SHARP and in external CKD cohorts. A user-friendly web interface (SHARP calculator, available at http://dismod.ndph.ox.ac.uk/kidneymodel/app/) which also includes projection of healthcare costs is freely available which facilitate model use. However, one of the limitations of the SHARP CKD-CVD model was that SHARP cohort [45] excluded patients with major coronary disease, whereas in routine clinical practice coronary heart disease is highly prevalent in CKD patients.

## 4. The Author's Experience

Portugal has the highest unadjusted incidence and prevalence of ESRD among European countries [46] and 67.7% of the incident dialysis patients, in 2015, were over 65 years with a mean age of prevalent patients of 66.7 years [47].

The Nephrology Department at Hospital de Santo António, Centro Hospitalar do Porto, conducted a retrospective cohort study of patients aged 65 years and over, referred to our Department, who started dialysis as their first RRT. This study aimed to identify elderly ESRD patients who have higher probability of death, early after starting dialysis, and develop a prognostic scoring model of 6-month mortality. This score was developed using data from a cohort of 360 patients who initiated dialysis between 2012 and 2015. Demographics and clinical variables were included as potential predictors. Multivariable adjusted logistic regression was used to determine the independent predictors of 6-month mortality. The *β*-coefficients from the final model (backward elimination) were used to generate point scores for calculating mortality risk. Then, our score was compared with others previously validated (Couchoud et al. [34] and Cohen et al. scores [42]).

In a univariate logistic analysis, the significant predictors of 6-month mortality were female gender, age > 75 years, ischemic heart disease, congestive heart failure, dysrhythmia, low albumin levels, unplanned dialysis, functional dependence, cognitive impairment, and being institutionalized. These candidate variables were included in a multivariable analysis and the regression *β*-coefficients from the final model were used to derive point scores to predict a patient's risk of dying in the first 6 months after starting dialysis. The final model for 6-month mortality risk included older age, female gender, ischemic heart disease, and low albumin levels* (articles submitted or in draft)*.

Our model does not seem to be weaker than other published scores (the area under the receiver operating characteristic curve (AUC) in our score, Couchoud et al. [34] and Cohen et al. [42] scores were 0.85, 0.73, and, 0.81, resp.).

This simple prediction score based on readily available clinical and laboratory data can be a practical and useful tool to assess short-term prognosis in elderly ESRD, although further research is needed to confirm and validate the use of this prognostic score.

## 5. Conclusions

Shared decision-making is a process of communication. It is particularly relevant when counselling elderly patients and their families on different RRT treatment options. This process may enable us to understand the advantages, limitations, and burdens, of the different treatment options, including conservative care.

Reliable, validated risk prediction models that correctly estimate risk of death after starting RRT may provide a more accurate perception of the desirability of starting dialysis and help in shared decision plan. Healthcare workers need to understand the applicability and limitations of these models so that they can be used appropriately.

In addition to the lack of external validation of the majority of the existing mortality scores, another important limitation is their inherent selection bias. Mostly, they were derived from patient populations who have initiated dialysis therapy and do not include those who are not selected for, or not accept, or do not survive to dialysis initiation. A score that evaluates older patients at the point of decision-making, rather than at the point of starting dialysis, would be more helpful.

Determining and communicating information prognosis for individual patients should be a part of clinical practice, and although the scores risk models cannot replace the clinical judgment, they are important instruments because they allow the patient to be aware of the future course of his disease and help physicians to guide clinical decision.
